# Resting heart rate is associated with the prevalence of chronic kidney disease in Korean adult: the Korean National Health and Nutrition Survey

**DOI:** 10.1186/s12889-024-17877-4

**Published:** 2024-02-05

**Authors:** Dong-Hyuk Park, Choon Hee Chung, Dong Hoon Lee, Eun Young Lee, Justin Y. Jeon

**Affiliations:** 1 Department of Sport Industry Studies, Yonsei University Sport Science Complex, 50 Yonsei-Ro, Seoul, Korea; 2 Exercise Medicine Center for Diabetes and Cancer Patients, ICONS, Yonsei University, Seoul, Korea; 3 Department of Internal Medicine, Yonsei University Wonju College of Medicine, Wonju, Korea; 4 Department of Nutrition, Harvard T. H. Chan School of Public Health, Boston, MA USA; 5 Department of Internal Medicine and Institute of Tissue Regeneration, Soonchunhyang University College of Medicine, Cheonan, BK21 FOUR Project Korea; 6 Cancer Prevention Center, Yonsei Cancer Center, Shinchon Severance Hospital, Yonsei University College of Medicine, Seoul, Korea

**Keywords:** Resting heart rate, Chronic kidney disease, Adults, Diabetes mellitus

## Abstract

**Background:**

Chronic kidney disease (CKD) poses a significant health challenge, yet early detection remains difficult. Resting heart rate (RHR) has been shown to be a reliable indicator of type 2 diabetes, prompting interest in its potential as an independent predictor of CKD. This study aimed to investigate the association between RHR and CKD prevalence, as well as explore potential interactions between RHR and other risk factors for CKD in a sample of 25,246 adults.

**Methods:**

Data from the Korean National Health and Nutrition Examination Survey (2011–2014) were utilized for this study, with 19,210 participants included after screening. Logistic regression analysis was employed to examine the relationship between RHR and CKD prevalence. Stratified analyses were conducted based on known risk factors for CKD.

**Results:**

Participants with an RHR ≥ 90 bpm exhibited a 2.07-fold [95% confidence interval (CI): 1.28–3.34] and 2.22-fold (95% CI: 1.42–3.48) higher prevalence of CKD in men and women, respectively, compared to those with an RHR < 60 bpm. The association between RHR and CKD prevalence was particularly pronounced in younger participants (40–59 years vs. ≥ 60 years), individuals with diabetes (yes vs. no), and those with a longer duration of diabetes (≥ 7 years vs. < 7 years).

**Conclusion:**

Elevated RHR was found to be significantly associated with a higher prevalence of CKD in both men and women, independent of demographic, lifestyle, and medical factors. These findings suggest that RHR could serve as a valuable predictor for undiagnosed CKD.

**Supplementary Information:**

The online version contains supplementary material available at 10.1186/s12889-024-17877-4.

## Background

Chronic kidney disease (CKD) is a substantial and escalating global health issue, characterized by a rapid rise in the number of people affected by CKD and end-stage renal disease (ESRD) [1]. CKD is one of the major contributors to all-cause mortality worldwide, accounting for 4.6% of total deaths [1]. Currently, more than 2.5 million people worldwide receive dialysis or kidney transplantation, which is expected to rise to 5.4 million by 2030 [2]. CKD is a significant social and personal concern, as it is linked with elevated mortality rates, the considerable financial burden for treatments, and reduced quality of life for those affected [3]. Early detection and awareness of CKD are crucial for improving disease prognosis, as it allows for prompt treatment and management. Unfortunately, less than a quarter of individuals with CKD in the US and fewer than 5% in South Korea are aware of their condition [4, 5]. Early intervention can result in better clinical outcomes and an enhanced quality of life for those affected by CKD.

Since most individuals with CKD also have diabetes and/or hypertension, a strong association of resting heart rate (RHR) with diabetes [6, 7] and hypertension [8] may suggest that RHR could serve as a predictive indicator for CKD. As a potentially modifiable risk factor, monitoring RHR in individuals with diabetes and/or hypertension could aid in the early detection and management of CKD, ultimately leading to improved patient outcomes. However, controversies exist regarding the association between RHR and cardiovascular disease (CVD) or renal disease outcomes in patients with CKD [9]. Furthermore, only a few studies investigated the relationship between RHR and CKD and they have focused on future health outcomes rather than the prevalence of CKD [10]. As a result, it is important to examine the relationship between RHR and the prevalence of CKD. Understanding this relationship could provide valuable information to aid in the early detection and management of CKD, potentially leading to improved patient outcomes. Therefore, the current study aimed to investigate the association between RHR and CKD prevalence in 25,246 Korean adults.

## Methods

### Study participants

The study utilized a large representative dataset from the Korean National Health and Nutrition Examination Study (KNHANES) conducted between 2011 and 2014. The initial sample included 25,246 participants, with 19,210 ultimately included in the final analysis. Participants were excluded if they had no data on RHR (*n* = 1,408), estimated glomerular filtration rate (eGFR; *n* = 3,295), albumin-to-creatinine ratio (ACR; *n* = 4,033), did not respond to CKD diagnosis (*n* = 1,663), were pregnant (*n* = 108), had a history of cancer (*n* = 878), had blood taken without fasting (*n* = 1,011), or had RHR of < 40 beats per minute (bpm, *n* = 9) or higher than 200 bpm (*n* = 1). Informed consent was obtained from all participants, and the study was approved by the Research Ethics Review Committee of the Korea Centers for Disease Control and Prevention.

### Data collection

The KNHANES data were collected using self-reported questionnaires or interviews with research staff [11]. The detailed procedures for the selection of households and methods of interviews have been described previously. Demographic, socioeconomic, and physical activity data were collected using self-reported questionnaires. Anthropometric (height, weight, and waist circumference) and metabolic risk factors (blood pressure, fasting glucose, HbA1c, and lipid profile) were measured or obtained from blood laboratory tests. Family history of diabetes, i.e., if either parent or any siblings had a history of diabetes, was recorded. Participants were asked if they were current, past, or never smoked. The frequency of drinking during the past year was assessed and categorized as less or more than once a month for the last year. Income was categorized into quartiles. The highest academic degree, including elementary school, middle school, high school, or college graduate or higher was surveyed. Sedentary time was also recorded in hours spent sitting on the usual day, and it was divided into two groups based on the median time. This information for diabetes, hypertension, and CVD was collected using a computer-assisted personal interviewing method.

### Resting heart rate

After a 5-min resting period in a seated position, RHR was measured for 15 s and then multiplied by 4 to calculate the heart rate per minute. If a participant’s heartbeat was irregular, with a slow pulse (< 10 beats per 15 s) or a rapid pulse (> 50 beats per 15 s), the heart rate was measured again for 1-min.

### Chronic kidney disease

Participants with CKD in our study were defined based on the diagnosis by a medical doctor or based on ACR ≥ 30 mg/g or eGFR < 60 mL/min/1.73 m^2^ [12]. The re-expressed four-variable Modification of Diet in Renal Disease study equations for standardized serum creatinine (Scr, mg/dL) is eGFR = 175 × standardized Scr^−1.154^ × age^−0.203^ × 1.212 [if black] × 0.742 [if female] [13].

### Diabetes

Participants with diabetes were defined by diagnosis by a medical doctor, oral hypoglycemic agents or insulin for the treatment of diabetes, or a fasting blood glucose level ≥ 126 mg/dL or HbA1c ≥ 6.5% [14]. Blood collection was conducted after fasting for at least 8 h at the mobile examination center through a screening investigation and was conducted and analyzed by experts consisting of nurses and clinical pathologists [11].

### Hypertension

Participants with hypertension were defined by diagnosis by a medical doctor, ongoing medications, or systolic blood pressure (SBP) ≥ 140 mmHg or diastolic blood pressure (DBP) ≥ 90 mmHg [11]. Blood pressure was measured after resting for 10 min and analyzed by experts comprising nurses and clinical pathologists.

### Cardiovascular disease

Participants with CVD (angina or myocardial infarction) were defined by diagnosis by a medical doctor, or ongoing medications.

### Statistical analysis

Descriptive analyses were used to examine the characteristics of the participants. To compare the differences in characteristics, we conducted an independent *t*-test for continuous variables and a *χ*^2^-test for categorical variables. For the main analyses, multivariable-adjusted logistic regression was used to estimate the odds ratios (ORs) and 95% confidence intervals (CIs) of the association between RHR as a quintile and the prevalence of CKD. To adjust for potential confounders, we included predefined covariates, including age (model 1), education, income, drinking, smoking, total physical activity, body mass index (BMI), family history of diabetes, family history of hypertension, menopausal status (model 2), diabetes, hypertension (model 3), fasting glucose level, HbA1c, SBP, DBP, myocardial infarction, angina and cardiovascular disease (model 4). Additional analyses were performed after adjusting for a potential mediator of the relationship between RHR and the prevalence of CKD.

For supplementary analyses, We also investigated the relationship between RHR quintiles and the prevalence of CKD. Lastly, we conducted a subgroup analysis to explore whether the associations between RHR and CKD prevalence would differ according to sociodemographic and lifestyle risk factors. To increase the robustness of our findings, we conducted a sensitivity analysis restricting participants without hypertension. All analyses were performed separately by sex, and all statistical analyses were performed using SPSS 25 version (IBM Co., Armonk, NY, USA).

## Results

### Baseline characteristics

Tables 1, 2, and 3 summarize participants’ characteristics by CKD prevalence and RHR. Generally, participants with CKD were older, less educated, and less physically active. The prevalence of diabetes was 36.0% (vs. 10.4% non-CKD) in participants with CKD. The prevalence of hypertension was 66.7% (vs. 27.7% non-CKD) in participants with CKD. Blood markers were significantly different between the CKD and non-CKD groups. Higher RHR was associated with younger age, lower BMI, lower physical activity level, a higher proportion of diabetes, and a higher proportion of ACR ≥ 30 mg/g. Participants with higher RHR are older, higher prevalence of CKD, and have higher ACR and eGFR. Participants with higher RHR are more likely to have diabetes, hypertension, and unawarded CKD.

**Table 1:** Characteristics of study participants

Participant (*n* = 19,210)	Men			Women			*p*-value
	Overall	Non-CKD	CKD	Overall	Non-CKD	CKD	
	*n* = 8,659	*n* = 7,690	*n* = 969	*n* = 10,551	*n* = 9,408	*n* = 1,143	
Resting heart rate, mean ± SE, n (%)	68.2 ± 0.1	68.0 ± 0.1	69.2 ± 0.3*	69.8 ± 0.1	69.7 ± 0.1	70.5 ± 0.3*	< 0.001
< 60 bpm	876 (10.1)	773 (10.1)	103 (10.6)	548 (5.2)	475 (5.0)	73 (6.4)	< 0.001
60-69 bpm	4,491 (51.9)	4,021 (52.3)	470 (48.5)	5,325 (50.5)	4,804 (51.1)	521 (45.6)	
70-79 bpm	2,071 (23.9)	1,857 (24.1)	214 (22.1)	2,844 (27.0)	2,536 (27.0)	308 (26.9)	
80-89 bpm	1,049 (12.1)	902 (11.7)	147 (15.2)	1,571 (14.9)	1,374 (14.6)	197 (17.2)	
≥ 90 bpm	172 (2.0)	137 (1.8)	35 (3.6)	263 (2.5)	219 (2.3)	44 (3.8)	
Age, mean ± SD, n (%)	49.5 ± 16.4	47.9 ± 16.0	44.7 ± 0.1*	50.6 ± 16.3	49.2 ± 15.8	62.2 ± 15.0*	< 0.001
BMI, mean ± SE, n (%)	24.1 ± 0.03	24.1 ± 0.04	24.7 ± 3.5*	23.5 ± 0.0	23.32 ± 0.0	24.67 ± 0.1*	< 0.001
ACR, mean ± SE, n (%)	20.8 ± 1.6	4.7 ± 0.1	148.8 ± 13.3*	19.3 ± 1.3	5.7 ± 0.1	131.2 ± 11.7*	< 0.001
≥ 30 mg/g	107 (1.2)	(0.0)	107 (11.0)	94 (0.9)	(0.0)	94 (8.2)	0.016
eGRF, mean ± SE, n (%)	85.4 ± 0.2	87.3 ± 0.2	70.9 ± 0.6*	89.2 ± 0.1	90.9 ± 0.1	75.5 ± 0.6*	< 0.001
< 60 ml/min/1.73m^2^	411 (4.7)	(0.0)	411 (42.4)	401 (3.8)	(0.0)	401 (35.1)	
Unawareness of CKD, n (%)	942 (10.9)	(0.0)	942 (97.2)	1,100 (10.4)	(0.0)	1,100 (96.2)	
Prevalence of diabetes, n (%)	1,272 (14.7)	863 (11.2)	409 (42.2)	1,261 (12.0)	909 (9.7)	352 (30.8)	< 0.001
Family history of diabetes, n (%)	1,210 (14.0)	1,102 (14.3)	108 (11.1)	1,557 (14.8)	1,444 (15.3)	113 (9.9)	
Diabetes duration							< 0.001
< 7 yr	375 (4.3)	259 (3.4)	116 (12.0)	362 (3.4)	278 (3.0)	84 (7.3)	
≥ 7 yr	388 (4.5)	220 (2.9)	168 (17.3)	386 (3.7)	240 (2.6)	146 (12.8)	
Missing	7,896 (91.2)	7,211 (93.8)	685 (70.7)	9,803 (92.9)	8,890 (94.5)	913 (79.9)	
Hypertension	3,013 (34.8)	2,343 (30.5)	670 (69.1)*	3,125 (29.6)	2,387 (25.4)	738 (64.6)*	< 0.001
Family history of hypertension, n (%)	2,444 (28.2)	2,221 (28.9)	223 (23.0)	3,263 (30.9)	3,000 (31.9)	263 (23.0)*	< 0.001
Hypertension duration							< 0.001
< 7 yr	922 (10.6)	720 (9.4)	202 (20.8)*	1,123 (10.6)	902 (9.6)	221 (19.3)*	
≥ 7 yr	818 (9.4)	540 (7.0)	278 (28.7)	1,125 (10.7)	784 (8.3)	341 (29.8)	
Missing	6,919 (79.9)	6,430 (83.6)	489 (50.5)	8,303 (78.7)	7,722 (82.1)	581 (50.8)	
Prevalence of CVD, n (%)	276 (3.2)	201 (2.6)	75 (7.7)	262 (2.5)	185 (2.0)	77 (6.7)	< 0.001
Alcohol, n (%)							< 0.001
Never	1,329 (15.3)	1,097 (14.3)	232 (23.9)*	3,782 (35.8)	3,211 (34.1)	571 (50.0)*	
< 10 g/day	1,616 (18.7)	1,468 (19.1)	148 (15.3)	3,763 (35.7)	3,440 (36.6)	323 (28.3)	
≥ 10 g/day	5,327 (61.5)	4,795 (62.4)	532 (54.9)	2,666 (25.3)	2,467 (26.2)	199 (17.4)	
Smoking, n(%)							< 0.001
Never	1,800 (20.8)	1,641 (21.3)	159 (16.4)*	9,191 (87.1)	8,195 (87.1)	996 (87.1)	
Past	3,174 (36.7)	2,716 (35.3)	458 (47.3)	497 (4.7)	450 (4.8)	47 (4.1)	
Current	3,296 (38.1)	3,003 (39.1)	293 (30.2)	515 (4.9)	468 (5.0)	47 (4.1)	
Education, n (%)							< 0.001
Elementary school	1,281 (14.8)	1,022 (13.3)	259 (26.7)*	3,040 (28.8)	2,408 (25.6)	632 (55.3)*	
Middle school	967 (11.2)	826 (10.7)	141 (14.6)	1,083 (10.3)	958 (10.2)	125 (10.9)	
High school	3,046 (35.2)	2,744 (35.7)	302 (31.2)	3,248 (30.8)	3,026 (32.2)	222 (19.4)	
Complemented Univ	2,916 (33.7)	2,706 (35.2)	210 (21.7)	2,758 (26.1)	2,654 (28.2)	104 (9.1)	
Income, n (%)							0.882
Low	2,018 (23.3)	1,763 (22.9)	255 (26.3) †	2,507 (23.8)	2,223 (23.6)	284 (24.8)	
Middle low	2,190 (25.3)	1,940 (25.2)	250 (25.8)	2,613 (24.8)	2,335 (24.8)	278 (24.3)	
Middle high	2,184 (25.2)	1,957 (25.4)	227 (23.4)	2,645 (25.1)	2,345 (24.9)	300 (26.2)	
High	2,198 (25.4)	1,974 (25.7)	224 (23.1)	2,697 (25.6)	2,428 (25.8)	269 (23.5)	
Physical activity, n (%)							< 0.001
< 150 min/week	4,819 (55.7)	4,239 (55.1)	580 (59.9) †	7,139 (67.7)	6,324 (67.2)	815 (71.3)*	
≥ 150 min/week	3,393 (39.2)	3,065 (39.9)	328 (33.8)	2,985 (28.3)	2,717 (28.9)	268 (23.4)	
Blood pressure, mmHg, mean ± SE							
SBP	120.8 ± 0.2	119.7 ± 0.2	129.3 ± 0.6*	117.0 ± 0.1	115.5 ± 0.1	129.3 ± 0.6*	< 0.001
DBP	77.9 ± 0.1	77.9 ± 0.1	78.2 ± 0.4†	73.5 ± 0.1	73.2 ± 0.1	75.6 ± 0.4†	< 0.001
Blood markers, mean ± SE							
Glucose, mg/dL	101.0 ± 0.2	99.2 ± 0.2	114.9 ± 1.1*	97.0 ± 0.2	95.6 ± 0.2	108.5 ± 1.0*	< 0.001
HbA1c, %	5.83 ± 0.01	5.76 ± 0.01	6.46 ± 0.04*	5.78 ± 0.01	5.73 ± 0.01	6.2 ± 0.03*	< 0.001
Total cholesterol, mg/dL	187.0 ± 0.4	187.2 ± 0.4	185.8 ± 1.2	191.4 ± 0.3	190.9 ± 0.4	196.0 ± 1.2*	< 0.001
Triglyceride, mg/dL	155.3 ± 1.4	152.3 ± 1.4	178.6 ± 4.6*	116.6 ± 0.8	112.7 ± 0.8	148.3 ± 2.9*	< 0.001
HDL, mg/dL	47.4 ± 0.1	47.7 ± 0.1	45.3 ± 0.4*	53.3 ± 0.1	53.8 ± 0.1	49.4 ± 0.3*	< 0.001
AST, IU/L	24.5 ± 0.2	24.2 ± 0.2	26.9 ± 0.5*	20.7 ± 0.1	20.4 ± 0.1	23.1 ± 0.4*	< 0.001
ALT, IU/L	25.9 ± 0.2	25.9 ± 0.3	26.0 ± 0.6*	17.9 ± 0.1	17.6 ± 0.1	20.2 ± 0.5*	< 0.001

Data are presented as mean ± *SD* or mean ± *SE* or N(%), *SD* Standard deviation, *SE* Standard Error, All variables were tested by ANCOVA (Analysis of covariance) or Chi-square test. ANCOVA was performed with age as a covariate. *p*-value significant differenct between men and women, *(*p* < 0.001) and †(*p* < 0.05 significant differenct between non-CKD and CKD, CKD Chronic Kidney Diesease, *eGFR* Estimated Glomerular Filtration Rate, *BMI* Body Mass Index, SBP Systolic Blood Pressure, *DBP* Dystolic Blood Pressure, *GOT* glutamic oxaloacetic transaminase, *GPT* glutamic pyruvic transaminase

**Table 2:** Characteristics of participants (Men)

Men *n* = 8,659	< 60 bpm	60–69 bpm	70–79 bpm	80–89 bpm	≥ 90 bpm	*p*-trend
	*n* = 876	*n* = 4,491	*n* = 2,071	*n* = 1,049	*n* = 172	
Age, mean ± SD, n (%)	54.2 ± 15.4	49.7 ± 16.1	47.9 ± 16.4	48.1 ± 17.6	49.1 ± 17.1	< 0.001
BMI, mean ± SE, n (%)	23.90 ± 0.09	24.24 ± 0.05	24.16 ± 0.07	23.85 ± 0.10	23.52 ± 0.28	< 0.001
Prevalence of CKD, n(%)	103 (11.8)	470 (10.5)	214 (10.3)	147 (14.0)	35 (20.3)	< 0.001
ACR, mean ± SE, n (%)	19.7 ± 3.7	19.6 ± 2.1	15.0 ± 1.6	35.4 ± 7.7	41.7 ± 11.4	< 0.001
≥ 30 mg/g	132 (15.1)	708 (15.8)	380 (18.3)	251 (23.9)	63 (36.6)	< 0.001
eGRF, mean ± SE, n (%)	82.1 ± 0.5	85.3 ± 0.2	86.4 ± 0.3	86.3 ± 0.5	89.3 ± 1.6	< 0.001
< 60 ml/min/1.73m^2^	51 (5.8)	207 (4.6)	74 (3.6)	66 (6.3)	13 (7.6)	< 0.001
Unawareness of CKD, n (%)	100 (11.4)	456 (10.2)	211 (10.2)	141 (13.4)	34 (19.8)	< 0.001
Prevalence of diabetes, n (%)	121 (13.8)	561 (12.5)	338 (16.3)	205 (19.5)	47 (27.3)	< 0.001
Family history of diabetes, n (%)	104 (11.9)	620 (13.8)	305 (14.7)	153 (14.6)	28 (16.3)	< 0.001
Diabetes duration						< 0.001
< 7 yr	37 (4.2)	154 (3.4)	109 (5.3)	66 (6.3)	9 (5.2)	
≥ 7 yr	36 (4.1)	175 (3.9)	94 (4.5)	60 (5.7)	23 (13.4)	
Missing	121 (13.8)	561 (12.5)	338 (16.3)	205 (19.5)	47 (27.3)	< 0.001
Hypertension	325 (37.1)	1,519 (33.8)	708 (34.2)	383 (36.5)	78 (45.3)	< 0.001
Family history of hypertension, n (%)	216 (24.7)	1,285 (28.6)	594 (28.7)	303 (28.9)	46 (26.7)	0.030
Hypertension duration						0.913
< 7 yr	140 (16.0)	456 (10.2)	202 (9.8)	103 (9.8)	21 (12.2)	
≥ 7 yr	91 (10.4)	427 (9.5)	186 (9.0)	100 (9.5)	14 (8.1)	
Missing	325 (37.1)	1,519 (33.8)	708 (34.2)	383 (36.5)	78 (45.3)	< 0.001
Prevalence of CVD, n (%)	49 (5.6)	146 (3.3)	52 (2.5)	27 (2.6)	2 (1.2)	< 0.001
Alcohol, n (%)						0.334
Never	155 (17.7)	680 (15.1)	305 (14.7)	154 (14.7)	35 (20.3)	
< 10 g/day	412 (47.0)	2,141 (47.7)	963 (46.5)	463 (44.1)	65 (37.8)	
≥ 10 g/day	268 (30.6)	1,484 (33.0)	710 (34.3)	373 (35.6)	64 (37.2)	
Smoking, n(%)						< 0.001
Never	165 (18.8)	929 (20.7)	444 (21.4)	229 (21.8)	33 (19.2)	
Past	388 (44.3)	1,697 (37.8)	689 (33.3)	343 (32.7)	57 (33.1)	
Current	283 (32.3)	1,676 (37.3)	844 (40.8)	419 (39.9)	74 (43.0)	
Education, n (%)						< 0.001
Elementary school	163 (18.6)	639 (14.2)	273 (13.2)	164 (15.6)	42 (24.4)	
Middle school	111 (12.7)	489 (10.9)	236 (11.4)	115 (11.0)	16 (9.3)	
High school	266 (30.4)	1,597 (35.6)	767 (37.0)	359 (34.2)	57 (33.1)	
Complemented Univ	290 (33.1)	1,546 (34.4)	684 (33.0)	349 (33.3)	47 (27.3)	
Income, n (%)						< 0.001
Low	199 (22.7)	988 (22.0)	503 (24.3)	272 (25.9)	56 (32.6)	
Middle low	221 (25.2)	1,167 (26.0)	494 (23.9)	253 (24.1)	55 (32.0)	
Middle high	227 (25.9)	1,139 (25.4)	538 (26.0)	249 (23.7)	31 (18.0)	
High	225 (25.7)	1,167 (26.0)	513 (24.8)	266 (25.4)	27 (15.7)	
Physical activity, n (%)						< 0.001
< 150 min/week	456 (52.1)	2,456 (54.7)	1,175 (56.7)	620 (59.1)	112 (65.1)	
≥ 150 min/week	374 (42.7)	1,814 (40.4)	789 (38.1)	366 (34.9)	50 (29.1)	
Blood pressure, mmHg, mean ± SE						
SBP	121.6 ± 0.5	120.4 ± 0.2	120.5 ± 0.3	121.5 ± 0.4	125.1 ± 1.4	0.003
DBP	74.6 ± 0.3	77.6 ± 0.2	79.1 ± 0.2	79.5 ± 0.3	79.8 ± 1.1	< 0.001
Blood markers, mean ± SE						
Glucose, mg/dL	98.8 ± 0.6	99.8 ± 0.3	102.0 ± 0.5	104.0 ± 0.8	111.6 ± 2.5	< 0.001
HbA1c, %	5.80 ± 0.02	5.79 ± 0.01	5.87 ± 0.02	5.94 ± 0.03	6.1 ± 0.08	< 0.001
Total cholesterol, mg/dL	184.2 ± 1.2	186.8 ± 0.5	188.7 ± 0.8	186.8 ± 1.1	189.2 ± 3.3	0.025
Triglyceride, mg/dL	132.3 ± 3.0	149.8 ± 1.7	165.1 ± 2.9	173.2 ± 5.0	187.0 ± 16.7	< 0.001
HDL, mg/dL	47.7 ± 0.4	47.3 ± 0.2	47.3 ± 0.2	47.4 ± 0.4	48.8 ± 1.0	0.700
AST, IU/L	23.8 ± 0.6	24.2 ± 0.2	24.6 ± 0.3	25.4 ± 0.5	28.9 ± 1.5	0.032
ALT, IU/L	23.7 ± 1.1	25.4 ± 0.3	26.8 ± 0.4	27.6 ± 0.7	28.7 ± 1.6	0.094

Data are presented as mean ± SD, mean ± SE, or n (%). SD, standard deviation; SE, standard error. All variables were tested by analysis of covariance (ANCOVA) or chi-square test. ANCOVA was performed with age as a covariate. Significant differences were found between quintiles of resting heart rate. *CKD* chronic kidney disease, *ACR* albumin-to-creatinine ratio, *eGFR* estimated glomerular filtration rate, *CVD* cardiovascular disease, *BMI* body mass index, *SBP* systolic blood pressure, *DBP* diastolic blood pressure AST Aspartate aminotransferase, *ALT* Alanine transaminase

**Table 3:** Characteristics of partipants (Women)

Women n = 10,551	< 60 bpm	60–69 bpm	70–79 bpm	80–89 bpm	≥ 90 bpm	*p*-trend
	*n* = 548	*n* = 5,325	*n* = 2,844	*n* = 1,571	*n* = 263	
Age, mean ± SD, n (%)	57.8 ± 13.4	51.9 ± 15.3	49.4 ± 16.7	47.0 ± 17.7	44.4 ± 19.4	< 0.001
BMI, mean ± SE, n (%)	24.0 ± 0.1	23.6 ± 0.0	23.4 ± 0.1	23.2 ± 0.1	22.7 ± 0.2	< 0.001
Prevalence of CKD, n(%)	73 (13.3)	521 (9.8)	308 (10.8)	197 (12.5)	44 (16.7)	< 0.001
ACR, mean ± SE, n (%)	16.3 ± 2.2	16.6 ± 1.5	20.5 ± 3.1	26.3 ± 4.5	27.1 ± 7.5	< 0.001
≥ 30 mg/g	82 (15.0)	737 (13.8)	451 (15.9)	293 (18.7)	74 (28.1)	< 0.001
eGRF, mean ± SE, n (%)	83.6 ± 0.7	88.4 ± 0.2	90.2 ± 0.3	91.0 ± 0.4	95.2 ± 1.2	< 0.001
< 60 ml/min/1.73m^2^	33 (6.0)	174 (3.3)	114 (4.0)	68 (4.3)	12 (4.6)	< 0.001
Unawareness of CKD, n (%)	68 (12.4)	499 (9.4)	299 (10.5)	190 (12.1)	44 (16.7)	< 0.001
Prevalence of diabetes, n (%)	63 (11.5)	590 (11.1)	335 (11.8)	236 (15.0)	37 (14.1)	< 0.001
Family history of diabetes, n (%)	47 (8.6)	763 (14.3)	463 (16.3)	245 (15.6)	39 (14.8)	< 0.001
Diabetes duration						< 0.001
< 7 yr	19 (3.5)	178 (3.3)	86 (3.0)	72 (4.6)	7 (2.7)	
≥ 7 yr	16 (2.9)	165 (3.1)	113 (4.0)	79 (5.0)	13 (4.9)	
Hypertension	222 (40.5)	1,608 (30.2)	789 (27.7)	423 (26.9)	83 (31.6)	< 0.001
Family history of hypertension, n (%)	156 (28.5)	1,681 (31.6)	876 (30.8)	466 (29.7)	84 (31.9)	0.030
Hypertension duration						0.913
< 7 yr	76 (13.9)	575 (10.8)	293 (10.3)	150 (9.5)	29 (11.0)	
≥ 7 yr	103 (18.8)	565 (10.6)	272 (9.6)	156 (9.9)	29 (11.0)	
Prevalence of CVD, n (%)	31 (5.7)	142 (2.7)	59 (2.1)	29 (1.8)	1 (0.4)	< 0.001
Alcohol, n (%)						0.334
Never	213 (38.9)	1,892 (35.5)	1,001 (35.2)	566 (36.0)	110 (41.8)	
< 10 g/day	292 (53.3)	2,890 (54.3)	1,543 (54.3)	843 (53.7)	123 (46.8)	
≥ 10 g/day	31 (5.7)	371 (7.0)	203 (7.1)	115 (7.3)	18 (6.8)	
Smoking, n(%)						< 0.001
Never	485 (88.5)	4,664 (87.6)	2,467 (86.7)	1,353 (86.1)	222 (84.4)	
Past	25 (4.6)	233 (4.4)	136 (4.8)	89 (5.7)	14 (5.3)	
Current	26 (4.7)	251 (4.7)	144 (5.1)	79 (5.0)	15 (5.7)	
Education, n (%)						< 0.001
Elementary school	227 (41.4)	1,568 (29.4)	779 (27.4)	407 (25.9)	59 (22.4)	
Middle school	80 (14.6)	584 (11.0)	269 (9.5)	135 (8.6)	15 (5.7)	
High school	155 (28.3)	1,632 (30.6)	900 (31.6)	463 (29.5)	98 (37.3)	
Complemented Univ	73 (13.3)	1,338 (25.1)	770 (27.1)	500 (31.8)	77 (29.3)	
Income, n (%)						< 0.001
Low	119 (21.7)	1,208 (22.7)	699 (24.6)	406 (25.8)	75 (28.5)	
Middle low	141 (25.7)	1,304 (24.5)	716 (25.2)	383 (24.4)	69 (26.2)	
Middle high	154 (28.1)	1,341 (25.2)	722 (25.4)	364 (23.2)	64 (24.3)	
High	131 (23.9)	1,422 (26.7)	685 (24.1)	404 (25.7)	55 (20.9)	
Physical activity, n (%)						< 0.001
< 150 min/week	355 (64.8)	3,516 (66.0)	1,987 (69.9)	1,087 (69.2)	194 (73.8)	
≥ 150 min/week	181 (33.0)	1,602 (30.1)	732 (25.7)	415 (26.4)	55 (20.9)	
Menopausal, n (%)						< 0.001
No	132 (24.1)	2,159 (40.5)	1,360 (47.8)	851 (54.2)	155 (58.9)	
Yes	404 (73.7)	2,957 (55.5)	1,357 (47.7)	651 (41.4)	94 (35.7)	
Blood pressure, mmHg, mean ± SE						
SBP	122.0 ± 0.7	117.7 ± 0.2	116.0 ± 0.3	114.7 ± 0.3	116.0 ± 0.9	0.003
DBP	71.6 ± 0.4	73.5 ± 0.1	73.6 ± 0.2	73.7 ± 0.2	74.3 ± 0.6	< 0.001
Blood markers, mean ± SE						
Glucose, mg/dL,	94.9 ± 0.7	96.2 ± 0.2	97.2 ± 0.4	99.8 ± 0.7	100.8 ± 1.5	< 0.001
HbA1c, %,	5.82 ± 0.02	5.77 ± 0.01	5.77 ± 0.01	5.83 ± 0.02	5.7 ± 0.05	< 0.001
Total cholestrol, mg/dL,	192.8 ± 1.5	192.0 ± 0.5	191.4 ± 0.7	189.6 ± 0.9	188.0 ± 2.3	0.025
Triglyceride, mg/dL,	116.5 ± 3.2	114.6 ± 1.0	118.1 ± 1.8	121.3 ± 2.1	112.6 ± 4.4	< 0.001
HDL, mg/dl	52.6 ± 0.5	53.3 ± 0.2	53.6 ± 0.2	53.2 ± 0.3	52.9 ± 0.8	0.700
AST, IU/L	21.7 ± 0.4	20.8 ± 0.1	20.5 ± 0.3	20.3 ± 0.3	20.0 ± 0.6	0.032
ALT, IU/L	18.6 ± 0.6	17.9 ± 0.2	17.8 ± 0.2	17.9 ± 0.4	17.4 ± 1.0	0.094

Data are presented as mean ± SD, mean ± SE, or n (%). SD, standard deviation; SE, standard error. All variables were tested by analysis of covariance (ANCOVA) or chi-square test. ANCOVA was performed with age as a covariate. Significant differences were found between quintiles of resting heart rate. *CKD* chronic kidney disease, *ACR* albumin-to-creatinine ratio, *eGFR* estimated glomerular filtration rate, *CVD* cardiovascular disease, *BMI* body mass index, *SBP* systolic blood pressure, *DBP* diastolic blood pressure, *AST* Aspartate aminotransferase, *ALT* Alanine transaminase

### Association between RHR and prevalence of CKD

Table 4 summarizes the association between RHR and CKD prevalence. In men, compared with participants whose RHR was < 60 bpm, participants whose RHR were at 80–89 bpm and ≥ 90 bpm showed 1.49 times (95% CI: 1.10–2.01) and 2.07 times (95% CI: 1.27–3.34) higher prevalence of CKD, respectively. In women, compared with participants whose RHR was < 60 bpm, participants whose RHR were between 80–89 bpm and ≥ 90 bpm showed 1.50 times (95% CI: 1.10–2.05) and 2.22 times (95% CI: 1.42–3.49) higher prevalence of CKD, respectively. The prevalence of CKD increases by 15% and 23% per 10 increments in RHR in men and women, respectively. Supplementary analyses which examined the association between the quintile of RHR and the prevalence of CKD showed similar results (Supplementary Table 4).

**Table 4:** Association of resting heart rate with the prevalence of chronic kidney disease

RHR (bpm)	< 60 bpm	60–69 bpm ORs(95%CIs)	70–79 bpm ORs(95%CIs)	80-89 bpm ORs(95%CIs)	≥ 90 bpm ORs(95%CIs)	p for trend	Per 10 increment in RHR ORs(95%CIs)
**Men**	103/876	470/4,491	214/2,071	147/1,049	35/172		
Model 1	1	1.11 (0.87, 1.40)	1.20 (0.93, 1.56)	1.64 (1.24, 2.18)	2.67 (1.69, 4.21)	< 0.001	1.21 (1.12, 1.30)
Model 2	1	1.13 (0.89, 1.44)	1.15 (0.88, 1.51)	1.54 (1.15, 2.07)	2.24 (1.39, 3.61)	< 0.001	1.16 (1.07, 1.25)
Model 3	1	1.11 (0.86, 1.42)	1.14 (0.85, 1.48)	1.49 (1.10, 2.01)	2.07 (1.28, 3.34)	< 0.001	1.15 (1.06, 1.24)
Model 4	1	1.11 (0.86, 1.42)	1.12 (0.85, 1.48)	1.49 (1.10, 2.01)	2.07 (1.28, 3.34)	< 0.001	1.14 (1.06, 1.24)
**Women**	73/548	521/35,325	308/2,844	137/1,571	44/263		
Model 1	1	0.91 (0.69, 1.19)	1.11 (0.84, 1.47)	1.45 (1.08, 1.96)	2.30 (1.49, 3.56)	< 0.001	1.24 (1.15, 1.33)
Model 2	1	0.92 (0.70, 1.21)	1.11 (0.83, 1.48)	1.37 (1.01, 1.86)	2.06 (1.32, 3.22)	< 0.001	1.20 (1.12, 1.29)
Model 3	1	0.96 (0.72, 1.26)	1.19 (0.83, 1.60)	1.51 (1.01, 2.06)	2.24 (1.43, 3.51)	< 0.001	1.24 (1.15, 1.33)
Model 4	1	0.95 (0.72, 1.26)	1.19 (0.89, 1.59)	1.50 (1.10, 2.04)	2.22 (1.42, 3.48)	< 0.001	1.23 (1.15, 1.33)

*RHR* resting heart rate, *ORs* odds ratios, *CIs* confidence intervals, Model 1 was adjusted for age. Model 2 was adjusted for model 1 variables + education, income, drinking, smoking, total physical activity, body mass index (Category), family history of diabetes, family history of hypertension, and menopause. Model 3 adjusted for model 2 variables + diabetes and hypertension. Model 4 was adjusted for model 3 variables + fasting glucose level, HbA1c, systolic blood pressure, diastolic blood pressure, and cardiovascular disease

Table 5 summarizes the stratified analyses on the association between RHR and the prevalence of CKD by potential effect modifiers. We found a positive association between the two regardless of the participant’s age, BMI, and lifestyle (alcohol, smoking, physical activity). The association between RHR and the prevalence of CKD was more evident among participants with younger age, diabetes, and longer diabetes duration. For every 10-bpm increment, the prevalence of CKD increased by 26% in participants aged < 40 years (vs. 19% in those aged 40–59 years and 12% in those aged ≥ 60 years), 32% in those with diabetes (vs. 12% in those without diabetes,), 38% in those with diabetes duration ≥ 7 years (vs. 15% in those with diabetes duration < 7 years), and 20% in those without hypertension (vs. 17% in those with hypertension).

**Table 5:** Stratified analyses of the association between resting heart rate and the prevalence of chronic kidney disease by potential effect modifiers

Chronic kidney disease	Number of case n(%)	Resting heart rate (bpm)						*p* for interaction
		< 60	60–69	70–79	80–89	≥ 90	10 bpm increment	
		Ref	ORs(95%CIs)	ORs(95%CIs)	ORs(95%CIs)	ORs(95%CIs)	ORs(95%CIs)	
Participants (*n* = 19,210)								
Age								0.022
< 40 yr	188/5,665 (3.3)	1	2.64 (0.63, 10.97)	2.88 (0.69, 12.08)	3.14 (0.74, 13.37)	7.11 (1.59, 31.71)	1.26 (1.08, 1.47)	
40–59 yr	545/7,412 (7.4)	1	1.09 (0.75, 1.60)	1.21 (0.81, 1.81)	1.74 (1.13, 2.67)	1.69 (0.85, 3.35)	1.19 (1.08, 1.32)	
≥ 60 yr	1379/6,133 (22.5)	1	0.99 (0.80, 1.23)	1.08 (0.85, 1.37)	1.32 (1.01, 1.72)	1.67 (1.08, 2.58)	1.12 (1.04, 1.19)	
Body mass index								0.423
< 23 kg/m^2^	685/8,352 (8.2)	1	0.96 (0.70, 1.32)	0.98 (0.69, 1.38)	1.38 (0.96, 1.99)	2.09 (1.28, 3.42)	1.17 (1.07, 1.28)	
23–24.9 kg/m^2^	1290/10,054 (12.8)	1	1.14 (0.79, 1.64)	1.37 (0.92, 2.04)	1.63 (1.06, 2.52)	2.30 (1.08, 4.91)	1.19 (1.07, 1.33)	
≥ 25 kg/m^2^	136/780 (17.4)	1	1.01 (0.76, 1.35)	1.22 (0.90, 1.66)	1.57 (1.12, 2.21)	2.21 (1.26, 3.84)	1.21 (1.11, 1.32)	
Alcohol								0.507
Never	803/5,111 (15.7)	1	0.93 (0.68, 1.26)	1.04 (0.75, 1.44)	1.33 (0.93, 1.90)	1.85 (1.09, 3.12)	1.15 (1.05, 1.25)	
≤ 1 day/month	847/9,735 (8.7)	1	0.96 (0.72, 1.26)	1.08 (0.79, 1.45)	1.32 (0.95, 1.84)	2.44 (1.44, 4.12)	1.17 (1.08, 1.27)	
≥ 2 days/month	355/3,637 (9.8)	1	2.09 (1.22, 3.59)	2.26 (1.29, 3.99)	3.30 (1.83, 5.96)	4.22 (1.88, 9.45)	1.30 (1.15, 1.48)	
Smoking								0.496
Never/previous	1660/14,662 (11.3)	1	1.07 (0.87, 1.31)	1.17 (0.94, 1.47)	1.48 (1.16, 1.89)	2.38 (1.65, 3.45)	1.18 (1.11, 1.25)	
Current	340/3,811 (8.9)	1	1.07 (0.67, 1.71)	1.31 (0.79, 2.14)	1.83 (1.07, 3.11)	2.36 (1.08, 5.15)	1.25 (1.10, 1.42)	
Physical activity								0.748
< 150 min/week	1395/11,958 (11.7)	1	0.89 (0.71, 1.12)	1.00 (0.78, 1.27)	1.35 (1.04, 1.75)	2.18 (1.47, 3.23)	1.18 (1.11, 1.26)	
≥ 150 min/week	596/6,378 (9.3)	1	1.52 (1.07, 2.17)	1.69 (1.15, 2.47)	1.90 (1.25, 2.89)	2.43 (1.25, 4.70)	1.17 (1.06, 1.29)	
Diabetes								0.035
No	1351/16,677 (8.1)	1	1.11 (0.89, 1.38)	1.12 (0.88, 1.42)	1.36 (1.05, 1.77)	2.14 (1.43, 3.21)	1.12 (1.05, 1.19)	
Yes	761/2,533 (30.0)	1	0.84 (0.59, 1.20)	1.25 (0.86, 1.82)	1.73 (1.17, 2.56)	2.13 (1.21, 3.74)	1.32 (1.20, 1.45)	
Diabetes duration								0.025
< 7 yr	200/737 (27.1)	1	1.02 (0.51, 2.04)	1.18 (0.63, 2.21)	1.75 (0.92, 3.32)	2.27 (1.05, 4.92)	1.15 (1.08, 1.21)	
≥ 7 yr	314/774 (40.6)	1	0.91 (0.49, 1.70)	1.31 (0.68, 2.54)	1.91 (0.95, 3.84)	2.88 (1.15, 7.22)	1.38 (1.18, 1.61)	
Hypertension								0.729
No	704/13,072 (5.4)	1	1.02 (0.75, 1.40)	1.14 (0.82, 1.59)	1.44 (1.01, 2.05)	2.92 (1.76, 4.85)	1.20 (1.10, 1.30)	
Yes	1408/6,138 (22.9)	1	1.03 (0.82, 1.30)	1.19 (0.93, 1.53)	1.54 (1.18, 2.02)	1.78 (1.18, 2.70)	1.17 (1.10, 1.25)	
Hypertension duration								0.065
< 7 yr	423/2,045 (20.7)	1	0.96 (0.66, 1.39)	0.91 (0.60, 1.38)	1.35 (0.86, 2.12)	1.16 (0.53, 2.52)	1.08 (0.96, 1.21)	
≥ 7 yr	619/1,943 (31.9)	1	1.02 (0.72, 1.45)	1.25 (0.85, 1.83)	1.61 (1.06, 2.46)	3.38 (1.66, 6.86)	1.25 (1.12, 1.39)	
Cardiovascular disease								0.877
No	1852/17,842 (10.4)	1	1.06 (0.87, 1.29)	1.14 (0.92, 1.41)	1.47 (1.17, 1.86)	2.29 (1.62, 3.22)	1.17 (1.11, 1.24)	
Yes	152/538 (28.3)	1	1.06 (0.57, 1.99)	2.24 (1.09, 4.57)	3.51 (1.52, 8.11)	6.91 (0.53, 89.90)	1.58 (1.24, 1.99)	

*ORs* odds ratios, *CIs* confidence intervals, Adjusted for age, sex, education, income, drinking, smoking, total physical activity, body mass index (category), family history of diabetes, family history of hypertension, menopausal, diabetes, hypertension, fasting glucose level, *HbA1c* systolic blood pressure, diastolic blood pressure, cardiovascular disease

## Discussion

We examined whether RHR was associated with CKD prevalence in a large sample of Korean adults; as hypothesized, we observed a significant association between the two. When participants were categorized according to every 10-bpm increment (< 60, 60–69, 70–79, 80–89, and ≥ 90 bpm). Compared with participants with RHR < 60 bpm, participants’ RHR between 80–89 bpm and ≥ 90 bpm showed a significantly higher prevalence of CKD in both men and women. Our main, sensitivity and supplementary analyses clearly showed a significant relationship between RHR and the prevalence of CKD.

We further studied whether these relationships exist when participants were subcategorized according to potential effect modifiers such as age, BMI, and comorbidities. Our subgroup analyses showed an increased prevalence of CKD when RHR increased regardless of age, BMI, alcohol consumption, smoking status, and prevalence of diabetes and hypertension. Significant interactions were also observed when participants were subcategorized according to the prevalence of diabetes, where the association between RHR and the prevalence of CKD was stronger. Given that diabetes is one of the most important factors contributing to CKD [15, 16], a stronger association between RHR and CKD in patients with diabetes added the value of RHR as a predictive variable that could be used for early detection of CKD in patients with diabetes [17].

Although the relationship between RHR and CKD is not fully understood, few studies have reported an association between RHR and kidney function [18]. Mao et al. [18]. studied the association between RHR and urinary ACR levels in 32,885 Chinese adults (middle-aged and older) and reported that participants whose RHR was > 87 bpm had a 17% higher prevalence of abnormal ACR levels. Bohm et al. [9]. also studied the association between RHR and renal disease outcomes among high-risk cardiovascular patients aged ≥ 55 years with coronary artery, peripheral vascular or cerebrovascular disease, or high-risk diabetes with end-organ damage. They reported significant associations between RHR and renal disease outcomes, including new microalbuminuria, doubling of serum creatinine, end-stage renal disease, and combined renal endpoint. Our study found results similar to those of previous studies that showed a significant association between RHR and CKD. However, the present study included adults older than 18 years, who represent the entire South Korean adult population, rather than middle-aged and patients aged > 55 years. Given that many people with CKD are unaware that they have CKD, which delays preventive intervention and treatment, our findings suggest the potential usefulness of RHR in predicting undiagnosed CKD.

To understand the association between RHR and the prevalence of CKD, it is important to determine whether high RHR is a risk factor or risk indicator of CKD. Higher RHR is known to be a risk factor for atherosclerosis [19] and CVD [20]. When adult male cynomolgus monkeys were fed an atherogenic high-cholesterol diet for 6 months, the animals that underwent sinoatrial node ablation had a lower degree of stenosis, which showed a direct relationship between RHR and stenosis. Although this study [19] did not assess renal arteries, atherosclerosis in the renal arteries could affect renal function. Furthermore, BEAUTIFUL (morbidity-mortality Evaluation of the *I*_f_ inhibitor ivabradine in patients with coronary disease and left-ventricular dysfunction) and SHIFT (Systolic Heart failure treatment with the *I*_f_ inhibitor ivabradine Trial) trials, a large randomized controlled trial, also showed that lowering RHR with medication could help in reducing major cardiovascular events, hospitalization in patients with stable coronary artery disease, and left ventricular systolic dysfunction, which is more evident when patients’ baseline RHR was above 70 or 75 bpm [20]. Follow-up studies that examined the heart rate lowering effect of ivabradine on renal function have reported a direct association between the increment of RHR and worsening of renal function; however, they did not observe any effect of ivabradine on renal function. Recently, Stanko et al. [21] reported that ivabradine ameliorates kidney fibrosis in L-NAME-induced hypertension, reduction of type I collagen volume, and enhanced vascular/perivascular type III collagen volume in mice. These studies demonstrated that high RHR could be a risk factor for atherosclerosis and cardiovascular disease outcomes, and lowering RHR could be beneficial for kidney function. However, it remains unclear whether lowering RHR directly affects kidney function in humans.

Although there is a lack of evidence on whether high RHR could result in higher CKD prevalence, ample evidence exists that higher RHR could be a risk indicator for CKD. Known risk factors for CKD include obesity [22], diabetes [15], and hypertension [23]. Interestingly, obesity [24], diabetes [6, 7, 25], and hypertension [8, 26] are significantly associated with RHR. Therefore, a higher RHR could reflect a higher prevalence of risk factors for CKD. Indeed, we observed significant differences in age, BMI, prevalence of diabetes, hypertension, smoking status, education, income, and level of physical activity when participants were categorized into quintiles. However, unlike previous studies [27], young age, diabetes, and diabetes duration were observed among those with higher RHR in our study, which suggests that a higher prevalence of CKD among participants with higher RHR was independent of age and BMI.

Because many people with CKD are unaware of their disease until their kidney dysfunction becomes irreversible, early diagnosis of CKD is important. Thus, our findings showed the utility of RHR in predicting undiagnosed CKD (early detection), if not alone, together with other risk factors of CKD. However, further prospective studies are needed to identify the role of RHR as a modifiable factor for prevention of incidence or progression of CKD.

Our study had some limitations. First, our study had a cross-sectional design; thus, it is difficult to examine the causal relationship between RHR and the risk of CKD. Second, there could be measurement errors in the assessment of RHR and CKD diagnosis. However, RHR was measured by professionally trained personnel, and all available CKD diagnoses were collected using detailed information from medical records, ACR, and eGFR. Third, although we comprehensively adjusted for various known risk factors, there could be residual confounding by unmeasured or unknown factors.

In conclusion, we clearly observed a significant positive association between RHR and CKD prevalence. A higher RHR was associated with an increased prevalence of CKD regardless of sex, age, and other potential confounding variables. The association between RHR and CKD prevalence is stronger in younger age, patients with diabetes, and diabetes diagnosis for more than 7 years, suggesting that a higher RHR could be used to predict undiagnosed CKD. Our findings showed that awareness of RHR could be used to screen personal health, including early detection of CKD.

### Supplementary Information

**Additional file 1: Supplementary 1.** Characteristics of study participants for men. **Supplementary 2.** Characteristics of study participants for women. **Supplementary 3.** Association between quintile of resting heart rate and the prevalence of chronic kidney disease. **Supplementary 4**. Stratified analyses on the association between quintile of resting heart rate and the prevalence of chronic kidney disease by potential effect modifiers. 

## Data Availability

The dataset can be downloaded from Korea National Health and Nutrition Examination Survey website (https://knhanes.kdca.go.kr/knhanes/sub04/sub04_04_01.do).
