# Supplementary material for: Resting heart rate is associated with the prevalence of chronic kidney disease in Korean adult: the Korean National Health and Nutrition Survey
Source: BMC Public Health. 2024 Feb 5;24:367. doi: 10.1186/s12889-024-17877-4 (PMC10840174; doi:10.1186/s12889-024-17877-4)
Supplement: Supplementary file 1 — Additional file 1: Supplementary 1. Characteristics of study participants for men. Supplementary 2. Characteristics of study participants for women. Supplementary 3. Association between quintile of resting heart rate and the prevalence of chronic kidney disease. Supplementary 4. Stratified analyses on the association between quintile of resting heart rate and the prevalence of chronic kidney disease by potential effect modifiers. [file 12889_2024_17877_MOESM1_ESM.docx]

| **Supplementary 1.** Characteristics of study participants for men | | | | | | | |
| --- | --- | --- | --- | --- | --- | --- | --- |
|  | Men | 1st quintile | 2nd quintile | 3rd quintile | 4th quintile | 5th quintile | *p*-trend |
|  |  | <61 bpm | 61–64 bpm | 65–68 bpm | 69–75 bpm | ≥76 bpm |  |
|  | n=8,659 | n=2,314 | n=1,598 | n=1,453 | n=1,325 | n=1,969 |  |
| Age, mean ± SD | 49.5 ± 16.4 | 52.0 ± 15.8 | 50.3 ± 16.2 | 48.1 ± 16.1 | 48.4 ± 16.6 | 47.8 ± 16.9 | <0.001 |
| BMI, mean ± SE | 24.12 ± 0.03 | 24.13 ± 0.06 | 24.12 ± 0.08 | 24.34 ± 0.08 | 24.12 ± 0.09 | 23.97±0.08 | <0.001 |
| Prevalence of CKD | 969 (11.2) | 259 (11.2) | 168 (10.5) | 146 (10.0) | 130 (9.8) | 266 (13.5) | 0.003 |
| ACR, mean ± SD, n(%) | 20.8 ± 1.6 | 21.8 ± 3.7 | 16.7 ± 2.0 | 19.2 ± 3.0 | 13.6 ± 1.6 | 29.1 ± 4.4 | <0.001 |
| ≥30 mg/g | 677 (7.8) | 158 (6.8) | 113 (7.1) | 112 (7.7) | 94 (7.1) | 199 (10.1) |  |
| eGFR, mean ± SD, n (%) | 85.4 ± 0.2 | 83.4 ± 0.3 | 85.0 ± 0.4 | 86.7 ± 0.4 | 86.0 ± 0.4 | 86.9 ± 0.4 | <0.001 |
| <60 ml/min/1.73m^2^ | 411 (4.7) | 134 (5.8) | 76 (4.8) | 48 (3.3) | 51 (3.8) | 102 (5.2) |  |
| Prevalence of diabetes, n (%) | 1,272 (14.7) | 272 (11.8) | 196 (12.3) | 214 (14.7) | 195 (14.7) | 395 (20.1) | <0.001 |
| Family history of diabetes, n (%) | 1,210 (14.0) | 293 (12.7) | 226 (14.1) | 205 (14.1) | 186 (14.0) | 300 (15.2) | 0.431 |
| Duration of diabetes, n (%) |  |  |  |  |  |  | 0.813 |
| <7 years | 375 (49.1) | 76 (48.4) | 55 (47.0) | 60 (46.9) | 62 (53.9) | 122 (49.6) |  |
| ≥7 years | 388 (50.9) | 81 (51.6) | 62 (53.0) | 68 (53.1) | 53 (46.1) | 124 (50.4) |  |
| Prevalence of hypertension, n (%) | 3,013 (34.8) | 823 (35.6) | 533 (33.4) | 487 (33.5) | 437 (33.0) | 733 (37.2) | 0.038 |
| Family history of hypertension, n (%) | 2,444 (28.2) | 636 (27.5) | 444 (27.8) | 420 (28.9) | 360 (27.2) | 584 (29.7) | 0.686 |
| Duration of hypertension, n (%) |  |  |  |  |  |  | 0.220 |
| <7 years | 922 (53.0) | 299 (57.0) | 155 (48.9) | 141 (52.0) | 130 (52.4) | 197 (52.0) |  |
| ≥7 years | 818 (47.0) | 226 (43.0) | 162 (51.1) | 130 (48.0) | 118 (47.6) | 182 (48.0) |  |
| Prevalence of CVD, n (%) | 276 (3.2) | 94 (4.1) | 52 (3.3) | 49 (3.4) | 37 (2.8) | 44 (2.2) | 0.023 |
| Alcohol, n (%) |  |  |  |  |  |  | 0.127 |
| Never | 1,329 (15.3) | 380 (16.4) | 256 (16.0) | 199 (13.7) | 199 (15.0) | 295 (15.0) |  |
| <10 g/day | 4,044 (46.7) | 441 (19.1) | 312 (19.5) | 250 (17.2) | 245 (18.5) | 368 (18.7) |  |
| ≥10 g/day | 2,899 (33.5) | 1,395 (60.3) | 964 (60.3) | 941 (64.8) | 826 (62.3) | 1,201 (61.0) |  |
| Smoking, n (%) |  |  |  |  |  |  | <0.001 |
| Never | 1,800 (20.8) | 432 (18.7) | 337 (21.1) | 324 (22.3) | 292 (22.0) | 415 (21.1) |  |
| Past | 3,174 (36.7) | 972 (42.0) | 592 (37.0) | 520 (35.8) | 446 (33.7) | 644 (32.7) |  |
| Current | 3,296 (38.1) | 812 (35.1) | 601 (37.6) | 546 (37.6) | 531 (40.1) | 806 (40.9) |  |
| Education, n (%) |  |  |  |  |  |  | 0.039 |
| Elementary school | 1,281 (14.8) | 380 (16.4) | 230 (14.4) | 191 (13.1) | 182 (13.7) | 298 (15.1) |  |
| Middle school | 967 (11.2) | 268 (11.6) | 182 (11.4) | 150 (10.3) | 147 (11.1) | 220 (11.2) |  |
| High school | 3,046 (35.2) | 763 (33.0) | 594 (37.2) | 505 (34.8) | 499 (37.7) | 685 (34.8) |  |
| Complemented university | 2,916 (33.7) | 788 (34.1) | 510 (31.9) | 538 (37.0) | 431 (32.5) | 649 (33.0) |  |
| Income, n (%) |  |  |  |  |  |  | <0.001 |
| Low | 2,018 (23.3) | 521 (22.5) | 363 (22.7) | 303 (20.9) | 315 (23.8) | 516 (26.2) |  |
| Middle low | 2,190 (25.3) | 593 (25.6) | 401 (25.1) | 394 (27.1) | 289 (21.8) | 513 (26.1) |  |
| Middle high | 2,184 (25.2) | 565 (24.4) | 416 (26.0) | 385 (26.5) | 377 (28.5) | 441 (22.4) |  |
| High | 2,198 (25.4) | 622 (26.9) | 407 (25.5) | 361 (24.8) | 327 (24.7) | 481 (24.4) |  |
| Physical activity, n (%) |  |  |  |  |  |  | 0.032 |
| <150 min/week | 4,819 (55.7) | 1,259 (54.4) | 867 (54.3) | 785 (54.0) | 763 (57.6) | 1,145 (58.2) |  |
| ≥150 min/week | 3,393 (39.2) | 938 (40.5) | 652 (40.8) | 597 (41.1) | 497 (37.5) | 709 (36.0) |  |
| SBP, mmHg, mean ± SE | 120.8 ± 0.2 | 121.5 ± 0.3 | 120.0 ± 0.4 | 120.0 ± 0.4 | 120.4 ± 0.4 | 121.5 ± 0.3 | <0.001 |
| DBP, mmHg, mean ± SE | 77.9 ± 0.1 | 76.4 ± 0.2 | 77.1 ± 0.3 | 78.3 ± 0.3 | 78.6 ± 0.3 | 79.7 ± 0.2 | <0.001 |
| Blood markers, mean ± SE |  |  |  |  |  |  |  |
| Glucose, mg/dL | 101.0 ± 0.2 | 98.5 ± 0.4 | 99.9 ± 0.5 | 101.1 ± 0.6 | 100.9 ± 0.6 | 104.7 ± 0.6 | <0.001 |
| HbA1c, % | 5.83 ± 0.01 | 5.77 ± 0.01 | 5.80 ± 0.02 | 5.82 ± 0.02 | 5.82 ± 0.02 | 6.00 ± 0.02 | <0.001 |
| Total cholesterol, mg/dL | 187.0 ± 0.4 | 186.3 ± 0.7 | 186.2 ± 0.8 | 186.6 ± 0.9 | 188.1 ± 1.0 | 188.1 ± 0.9 | 0.187 |
| Triglyceride, mg/dL | 155.3 ± 1.4 | 141.7 ± 2.1 | 146.7 ± 2.7 | 155.1 ± 3.2 | 159.4 ± 3.4 | 175.3 ± 3.6 | <0.001 |
| HDL, mg/dL | 47.4 ± 0.1 | 47.7 ± 0.2 | 46.9 ± 0.3 | 47.3 ± 0.3 | 47.5 ± 0.3 | 47.4 ± 0.3 | <0.001 |
| AST, IU/L | 24.5 ± 0.2 | 23.9 ± 0.3 | 23.9 ± 0.3 | 24.8 ± 0.5 | 24.0 ± 0.4 | 25.8 ± 0.4 | <0.001 |
| ALT, IU/L | 25.9 ± 0.2 | 24.3 ± 0.5 | 25.0 ± 0.4 | 26.7 ± 0.7 | 25.7 ± 0.5 | 28.1 ± 0.5 | <0.001 |
| Data are presented as mean ± SD, mean ± SE, or n (%). SD, standard deviation; SE, standard error. All variables were tested by analysis of covariance (ANCOVA) or chi-square test. ANCOVA was performed with age as a covariate. Significant differences were found between quintiles of resting heart rate. CKD, chronic kidney disease; ACR, albumin-to-creatinine ratio; eGFR, estimated glomerular filtration rate; CVD, cardiovascular disease; BMI, body mass index; SBP, systolic blood pressure; DBP, diastolic blood pressure; AST, Aspartate aminotransferase; ALT, Alanine transaminase. | | | | | | | |

| **Supplementary 2.** Characteristics of study participants for women | | | | | | | |
| --- | --- | --- | --- | --- | --- | --- | --- |
|  | Women | 1st quintile | 2nd quintile | 3rd quintile | 4th quintile | 5th quintile | *p*-trend |
|  |  | <64 bpm | 64–67 bpm | 68–70 bpm | 71–75 bpm | ≥76 bpm |  |
|  | n=10,551 | n=2,027 | n=1,942 | n=1,905 | n=1,753 | n=2,924 |  |
| Age, mean ± SD | 50.6 ± 16.3 | 54.8 ± 14.3 | 51.9 ± 15.4 | 50.4 ± 15.6 | 50.3 ± 16.5 | 47.1 ± 17.6 | <0.001 |
| BMI, mean ± SE | 23.46 ± 0.03 | 23.75 ± 0.07 | 23.60 ± 0.08 | 23.48 ± 0.08 | 23.48 ± 0.08 | 23.16 ± 0.07 | <0.001 |
| Prevalence of CKD | 1,143 (10.8) | 217 (10.7) | 204 (10.5) | 173 (9.1) | 192 (11.0) | 357 (12.2) | 0.017 |
| ACR, mean ± SD, n(%) | 19.3 ± 1.3 | 13.3 ± 1.2 | 16.1 ± 1.6 | 20.4 ± 3.5 | 19.3 ± 3.1 | 25.0 ± 3.5 | <0.001 |
| ≥30 mg/g | 855 (8.1) | 148 (7.3) | 154 (8.0) | 135 (7.1) | 136 (7.8) | 282 (9.6) | 0.002 |
| eGFR, mean ± SD, n (%) | 89.2 ± 0.1 | 85.9 ± 0.3 | 88.4 ± 0.3 | 89.7 ± 0.3 | 89.8 ± 0.4 | 91.4 ± 0.3 | <0.001 |
| <60 ml/min/1.73m^2^ | 401 (3.8) | 86 (4.2) | 71 (3.7) | 50 (2.6) | 71 (4.1) | 123 (4.2) | 0.040 |
| Prevalence of diabetes, n (%) | 1,261 (12.0) | 219 (10.8) | 228 (11.7) | 207 (10.9) | 199 (11.4) | 408 (14.0) | 0.002 |
| Family history of diabetes, n (%) | 1,557 (14.8) | 263 (13.0) | 263 (13.5) | 284 (14.9) | 272 (15.5) | 475 (16.2) | <0.001 |
| Duration of diabetes, n (%) |  |  |  |  |  |  | 0.100 |
| <7 years | 362 (48.4) | 70 (53.8) | 70 (50.0) | 58 (53.2) | 45 (38.1) | 119 (47.4) |  |
| ≥7 years | 386 (51.6) | 60 (46.2) | 70 (50.0) | 51 (46.8) | 73 (61.9) | 132 (52.6) |  |
| Prevalence of hypertension, n (%) | 3,125 (29.6) | 715 (35.3) | 615 (31.7) | 501 (26.3) | 505 (28.8) | 789 (27.0) | 0.038 |
| Family history of hypertension, n (%) | 3,263 (30.9) | 642 (31.7) | 614 (31.6) | 581 (30.5) | 528 (30.1) | 898 (30.7) | 0.005 |
| Duration of hypertension, n (%) |  |  |  |  |  |  | 0.041 |
| <7 years | 1,123 (50.0) | 243 (45.3) | 242 (55.0) | 166 (48.3) | 179 (49.9) | 293 (51.5) |  |
| ≥7 years | 1,125 (50.0) | 293 (54.7) | 198 (45.0) | 178 (51.7) | 180 (50.1) | 276 (48.5) |  |
| Prevalence of CVD, n (%) | 262 (2.5) | 77 (3.8) | 56 (2.9) | 40 (2.1) | 34 (1.9) | 55 (1.9) | 0.003 |
| Alcohol, n (%) |  |  |  |  |  |  | 0.726 |
| Never | 3,782 (35.8) | 755 (37.2) | 681 (35.1) | 670 (35.2) | 608 (34.7) | 1,068 (36.5) |  |
| <10 g/day | 5,691 (53.9) | 1,093 (53.9) | 1,052 (54.2) | 1,037 (54.4) | 954 (54.4) | 1,555 (53.2) |  |
| ≥10 g/day | 738 (7.0) | 121 (6.0) | 146 (7.5) | 135 (7.1) | 130 (7.4) | 206 (7.0) |  |
| Smoking, n (%) |  |  |  |  |  |  | 0.561 |
| Never | 9,191 (87.1) | 1,785 (88.1) | 1,695 (87.3) | 1,670 (87.7) | 1,519 (86.7) | 2,522 (86.3) |  |
| Past | 497 (4.7) | 97 (4.8) | 77 (4.0) | 84 (4.4) | 83 (4.7) | 156 (5.3) |  |
| Current | 515 (4.9) | 86 (4.2) | 105 (5.4) | 86 (4.5) | 90 (5.1) | 148 (5.1) |  |
| Education, n (%) |  |  |  |  |  |  | <0.001 |
| Elementary school | 3,040 (28.8) | 682 (33.6) | 596 (30.7) | 518 (27.2) | 502 (28.6) | 742 (25.4) |  |
| Middle school | 1,083 (10.3) | 255 (12.6) | 209 (10.8) | 200 (10.5) | 176 (10.0) | 243 (8.3) |  |
| High school | 3,248 (30.8) | 582 (28.7) | 580 (29.9) | 625 (32.8) | 540 (30.8) | 921 (31.5) |  |
| Complemented university | 2,758 (26.1) | 436 (21.5) | 489 (25.2) | 486 (25.5) | 458 (26.1) | 889 (30.4) |  |
| Income, n (%) |  |  |  |  |  |  | 0.048 |
| Low | 2,507 (23.8) | 415 (20.5) | 479 (24.7) | 433 (22.7) | 435 (24.8) | 745 (25.5) |  |
| Middle low | 2,613 (24.8) | 505 (24.9) | 462 (23.8) | 479 (25.1) | 438 (25.0) | 729 (24.9) |  |
| Middle high | 2,645 (25.1) | 541 (54.1) | 463 (23.8) | 491 (25.8) | 437 (24.9) | 713 (24.4) |  |
| High | 2,697 (25.6) | 549 (54.9) | 519 (26.7) | 485 (25.5) | 431 (24.6) | 713 (24.4) |  |
| Physical activity, n (%) |  |  |  |  |  |  | <0.001 |
| <150 min/week | 7,139 (67.7) | 1,319 (65.1) | 1,284 (66.1) | 1,269 (66.6) | 1,220 (69.6) | 2,047 (70.0) |  |
| ≥150 min/week | 2,985 (28.3) | 636 (31.4) | 589 (30.3) | 558 (29.3) | 457 (26.1) | 745 (25.5) |  |
| SBP, mmHg, mean ± SE | 117.0 ± 0.1 | 120.1 ± 0.4 | 117.7 ± 0.3 | 116.3 ± 0.3 | 116.9 ± 0.4 | 114.8 ± 0.3 | <0.001 |
| DBP, mmHg, mean ± SE | 73.5 ± 0.1 | 73.0 ± 0.2 | 73.4 ± 0.2 | 73.7 ± 0.2 | 73.7 ± 0.2 | 73.7 ± 0.2 | <0.001 |
| Blood markers, mean ± SE |  |  |  |  |  |  |  |
| Glucose, mg/dL | 97.0 ± 0.2 | 95.5 ± 0.4 | 96.8 ± 0.4 | 95.9 ± 0.4 | 97.3 ± 0.5 | 98.9 ± 0.4 | <0.001 |
| HbA1c, % | 5.78 ± 0.01 | 5.78 ± 0.01 | 5.80 ± 0.02 | 5.75 ± 0.02 | 5.77 ± 0.02 | 5.80 ± 0.02 | <0.001 |
| Total cholesterol, mg/dL | 191.4 ± 0.3 | 193.5 ± 0.8 | 191.0 ± 0.8 | 191.6 ± 0.8 | 192.9 ± 0.9 | 189.3 ± 0.7 | <0.001 |
| Triglyceride, mg/dL | 116.6 ± 0.8 | 114.9 ± 1.6 | 114.1 ± 1.6 | 115.3 ± 1.7 | 119.6 ± 2.5 | 118.4 ± 1.4 | <0.001 |
| HDL, mg/dL | 53.3 ± 0.1 | 53.3 ± 0.3 | 53.1 ± 0.3 | 53.2 ± 0.3 | 53.5 ± 0.3 | 53.4 ± 0.2 | <0.001 |
| AST, IU/L | 20.7 ± 0.1 | 21.4 ± 0.2 | 20.6 ± 0.2 | 20.7 ± 0.2 | 20.5 ± 0.4 | 20.3 ± 0.2 | <0.001 |
| ALT, IU/L | 17.9 ± 0.1 | 18.3 ± 0.3 | 17.7 ± 0.3 | 18.0 ± 0.4 | 17.6 ± 0.3 | 17.9 ± 0.3 | <0.001 |
| Data are presented as mean ± SD, mean ± SE, or n (%). SD, standard deviation; SE, standard error. All variables were tested by analysis of covariance (ANCOVA) or chi-square test. ANCOVA was performed with age as a covariate. Significant differences were found between quintiles of resting heart rate. CKD, chronic kidney disease; ACR, albumin-to-creatinine ratio; eGFR, estimated glomerular filtration rate; CVD, cardiovascular disease; BMI, body mass index; SBP, systolic blood pressure; DBP, diastolic blood pressure; AST, Aspartate aminotransferase; ALT, Alanine transaminase. | | | | | | | |

|  | | | | | | | |
| --- | --- | --- | --- | --- | --- | --- | --- |
| **Supplementary 3.** Association between resting heart rate and the prevalence of chronic kidney disease | | | | | | | |
| RHR (bpm) | 1^st^ quintile | 2^nd^ quintile | 3^rd^ quintile | 4^th^ quintile | 5^th^ quintile | *p* for trend | Per 5 increment in RHR |
|  | M: <61 bpm  W: <64 bpm | M: 61–64 bpm  W: 64–67 bpm | M: 65–68 bpm  W: 68–70 bpm | M: 69–75 bpm  W: 71–75 bpm | M: ≥76 bpm  W: ≥76 bpm |  |  |
| Men  (969/8,659) | 259/2,314 | 168/1,598 | 146/1,453 | 130/1,325 | 266/1,969 |  |  |
|  |  |  |  |  |  |  |  |
| Crude | 1 | 0.93 (0.76, 1.14) | 0.89 (0.72, 1.10) | 0.86 (0.69, 1.08) | 1.24 (1.03, 1.49) | <0.001 | 1.06 (1.02, 1.10) |
| Model 1 | 1 | 1.00 (0.81, 1.24) | 1.09 (0.87, 1.37) | 1.02 (0.81, 1.29) | 1.55 (1.28, 1.88) | <0.001 | 1.10 (1.06, 1.15) |
| Model 2 | 1 | 0.99 (0.79, 1.24) | 1.02 (0.81, 1.28) | 0.97 (0.77, 1.24) | 1.36 (1.11, 1.67) | <0.001 | 1.10 (1.06, 1.15) |
| Model 3 | 1 | 0.99 (0.79, 1.24) | 1.02 (0.81, 1.28) | 0.97 (0.77, 1.24) | 1.36 (1.11, 1.67) | <0.001 | 1.08 (1.04, 1.12) |
| Model 4 | 1 | 1.00 (0.80, 1.25) | 1.03 (0.81, 1.30) | 0.97 (0.76, 1.23) | 1.35 (1.10, 1.65) | <0.001 | 1.07 (1.03, 1.12) |
| Women (1,143/10,551) | 217/2,027 | 204/1,942 | 173/1,905 | 192/1,753 | 357/2,924 |  |  |
|  |  |  |  |  |  |  |  |
| Crude | 1 | 0.98 (0.80, 1.20) | 0.83 (0.68, 1.03) | 1.03 (0.84, 1.26) | 1.16 (0.97, 1.39) | <0.001 | 1.05 (1.01, 1.08) |
| Model 1 | 1 | 1.11 (0.90, 1.36) | 1.01 (0.81, 1.25) | 1.22 (0.99, 1.51) | 1.59 (1.32, 1.92) | <0.001 | 1.11 (1.07, 1.15) |
| Model 2 | 1 | 1.09 (0.88, 1.35) | 1.05 (0.84, 1.31) | 1.23 (0.99, 1.52) | 1.50 (1.24, 1.82) | <0.001 | 1.11 (1.07, 1.15) |
| Model 3 | 1 | 1.09 (0.88, 1.35) | 1.05 (0.84, 1.31) | 1.23 (0.99, 1.52) | 1.50 (1.24, 1.82) | <0.001 | 1.09 (1.06, 1.13) |
| Model 4 | 1 | 1.13 (0.91, 1.40) | 1.10 (0.88, 1.38) | 1.29 (1.04, 1.61) | 1.64 (1.35, 2.00) | <0.001 | 1.11 (1.07, 1.15) |
| RHR, resting heart rate; OR, odds ratio; 95% CI, 95% confidence interval. Model 1 was adjusted for age. Model 2 was adjusted for model 1 variables + education, income, drinking, smoking, total physical activity, body mass index (Category), family history of diabetes, family history of hypertension, and menopause. Model 3 adjusted for model 2 variables + diabetes and hypertension. Model 4 was adjusted for model 3 variables + fasting glucose level, HbA1c, systolic blood pressure, diastolic blood pressure, and cardiovascular disease. | | | | | | | |

| **Supplementary 4**. Stratified analyses on the association between resting heart rate and the prevalence of chronic kidney disease by potential effect modifiers | | | | | | | |
| --- | --- | --- | --- | --- | --- | --- | --- |
| Chronic kidney disease | Number of case  n (%) | Resting heart rate (bpm) | | | | | |
|  |  | 1st quintile | 2nd quintile | 3rd quintile | 4th quintile | 5th quintile | Per 5 increment in RHR |
|  |  | Ref | OR (95% CI) | OR (95% CI) | OR (95% CI) | OR (95% CI) | OR (95% CI) |
| Participants (n=19,210) |  |  |  |  |  |  |  |
| Age |  |  |  |  |  |  |  |
| <40 yr | 188/5,665 (3.3) | 1 | 1.61 (0.87, 2.98) | 1.20 (0.64, 2.23) | 1.40 (0.76, 2.57) | 1.77 (1.03, 3.05) | 1.12 (1.03, 1.21) |
| 40-59 yr | 545/7,412 (7.4) | 1 | 0.96 (0.71, 1.29) | 1.30 (0.97, 1.73) | 1.05 (0.77, 1.44) | 1.60 (1.23, 2.09) | 1.11 (1.05, 1.17) |
| ≥60 yr | 1379/6,133 (22.5) | 1 | 1.06 (0.87, 1.28) | 0.96 (0.78, 1.18) | 1.10 (0.90, 1.35) | 1.29 (1.07, 1.54) | 1.06 (1.02, 1.10) |
| Body mass index |  |  |  |  |  |  |  |
| <23 kg/m^2^ | 685/8,352 (8.2) | 1 | 1.08 (0.83, 1.41) | 0.91 (0.68, 1.21) | 0.97 (0.73, 1.29) | 1.39 (1.09, 1.76) | 1.08 (1.04, 1.13) |
| 23–24.9 kg/m^2^ | 1290/10,054 (12.8) | 1 | 1.14 (0.84, 1.56) | 1.12 (0.81, 1.55) | 1.33 (0.96, 1.84) | 1.52 (1.13, 2.04) | 1.10 (1.04, 1.16) |
| ≥25 kg/m^2^ | 136/780 (17.4) | 1 | 1.00 (0.78, 1.27) | 1.15 (0.91, 1.47) | 1.16 (0.91, 1.49) | 1.61 (1.29, 2.00) | 1.11 (1.06, 1.16) |
| Alcohol |  |  |  |  |  |  |  |
| Never | 803/5,111 (15.7) | 1 | 1.23 (0.95, 1.59) | 1.11 (0.84, 1.46) | 1.22 (0.93, 1.60) | 1.50 (1.18, 1.90) | 1.08 (1.03, 1.12) |
| <10 g/day | 847/9,735 (8.7) | 1 | 0.98 (0.77, 1.23) | 1.08 (0.84, 1.37) | 1.06 (0.83, 1.36) | 1.42 (1.14, 1.77) | 1.09 (1.04, 1.13) |
| ≥10 g/day | 355/3,637 (9.8) | 1 | 1.05 (0.71, 1.54) | 1.08 (0.73, 1.59) | 1.09 (0.74, 1.62) | 1.70 (1.22, 2.36) | 1.15 (1.08, 1.22) |
| Smoking |  |  |  |  |  |  |  |
| Never/previous | 1660/14,662 (11.3) | 1 | 1.12 (0.95, 1.34) | 1.20 (1.00, 1.43) | 1.21 (1.01, 1.45) | 1.49 (1.27, 1.75) | 1.09 (1.06, 1.12) |
| Current | 340/3,811 (8.9) | 1 | 0.86 (0.58, 1.27) | 0.61 (0.39, 0.95) | 0.81 (0.53, 1.21) | 1.52 (1.09, 2.11) | 1.12 (1.05, 1.20) |
| Physical activity |  |  |  |  |  |  |  |
| <150 min/week | 1395/11,958 (11.7) | 1 | 0.97 (0.80, 1.18) | 1.01 (0.82, 1.23) | 1.05 (0.86, 1.29) | 1.44 (1.21, 1.71) | 1.09 (1.06, 1.13) |
| ≥150 min/week | 596/6,378 (9.3) | 1 | 1.28 (0.97, 1.68) | 1.16 (0.86, 1.55) | 1.21 (0.89, 1.64) | 1.53 (1.18, 1.98) | 1.08 (1.03, 1.14) |
| Diabetes |  |  |  |  |  |  |  |
| No | 1351/16,677 (8.1) | 1 | 1.05 (0.88, 1.25) | 1.06 (0.88, 1.28) | 1.05 (0.87, 1.27) | 1.25 (1.06, 1.48) | 1.06 (1.02, 1.09) |
| Yes | 761/2,533 (30.0) | 1 | 1.14 (0.84, 1.55) | 1.07 (0.78, 1.47) | 1.35 (0.99, 1.85) | 2.11 (1.61, 2.75) | 1.16 (1.11, 1.22) |
| Diabetes duration |  |  |  |  |  |  |  |
| <7 years | 200/737 (27.1) | 1 | 1.01 (0.85, 1.20) | 1.07 (0.90, 1.27) | 1.13 (0.95, 1.35) | 1.36 (1.16, 1.58) | 0.00 (0.00, 0.00) |
| ≥7 years | 314/774 (40.6) | 1 | 1.53 (0.91, 2.58) | 1.19 (0.69, 2.06) | 1.41 (0.82, 2.41) | 2.65 (1.66, 4.23) | 1.18 (1.09, 1.28) |
| Hypertension |  |  |  |  |  |  |  |
| No | 704/13,072 (5.4) | 1 | 1.15 (0.90, 1.48) | 1.04 (0.80, 1.36) | 1.23 (0.95, 1.60) | 1.45 (1.15, 1.82) | 1.09 (1.05, 1.14) |
| Yes | 1408/6,138 (22.9) | 1 | 1.01 (0.83, 1.23) | 1.07 (0.87, 1.32) | 1.08 (0.87, 1.32) | 1.51 (1.27, 1.81) | 1.09 (1.06, 1.13) |
| Hypertension duration |  |  |  |  |  |  |  |
| <7 years | 423/2,045 (20.7) | 1 | 0.95 (0.67, 1.33) | 1.10 (0.77, 1.58) | 0.88 (0.61, 1.29) | 1.25 (0.91, 1.71) | 1.04 (0.98, 1.11) |
| ≥7 years | 619/1,943 (31.9) | 1 | 1.03 (0.76, 1.40) | 0.81 (0.58, 1.13) | 1.07 (0.78, 1.48) | 1.59 (1.20, 2.11) | 1.12 (1.06, 1.18) |
| CVD |  |  |  |  |  |  |  |
| No | 1852/17,842 (10.4) | 1 | 1.09 (0.92, 1.28) | 1.10 (0.93, 1.30) | 1.09 (0.92, 1.29) | 1.46 (1.25, 1.69) | 1.09 (1.06, 1.12) |
| Yes | 152/538 (28.3) | 1 | 0.86 (0.46, 1.62) | 0.71 (0.36, 1.41) | 1.78 (0.91, 3.48) | 2.62 (1.43, 4.81) | 1.25 (1.11, 1.41) |
| OR = Odds Ratio, 95%CI = 95% Confidence Interval, Adjusted for age, sex, education, income, drinking, smoking, total physical activity, body mass index (category), family history of diabetes, family history of hypertension, menopausal, diabetes, hypertension, fasting glucose level, HbA1c, systolic blood pressure, diastolic blood pressure, cardiovascular disease | | | | | | | |
